# Essential *Acidovorax citrulli* Virulence Gene *hrpE* Activates Host Immune Response against Pathogen

**DOI:** 10.3390/ijms23169144

**Published:** 2022-08-15

**Authors:** Weiqin Ji, Mei Zhao, Nuoya Fei, Linlin Yang, Pei Qiao, Ron Walcott, Yuwen Yang, Tingchang Zhao

**Affiliations:** 1 State Key Laboratory for Biology of Plant Diseases and Insect Pests, Institute of Plant Protection, Chinese Academy of Agricultural Sciences, Beijing 100193, China; 2 Department of Plant Pathology, College of Plant Protection, China Agricultural University, Beijing 100193, China; 3 Department of Plant Pathology, University of Georgia, Athens, GA 30602, USA

**Keywords:** BFB, HrpE, pathogenesis, host immune response, biological function

## Abstract

Bacterial fruit blotch (BFB) caused by *Acidovorax citrulli* (Ac) is a devastating watermelon disease that severely impacts the global watermelon industry. Like other Gram-negative bacteria, the type three secretion system (T3SS) is the main pathogenicity factor of *A. citrulli*. The T3SS apparatus gene *hrpE* codes for the Hrp pilus and serves as a conduit to secret effector proteins into host cells. In this study, we found that the deletion of *hrpE* in *A. citrulli* results in the loss of pathogenicity on hosts and the hypersensitive response on non-hosts. In addition, the *A. citrulli hrpE* mutant showed a reduction in in vitro growth, *in planta* colonization, swimming and twitching motility, and displayed increases in biofilm formation ability compared to the wild type. However, when HrpE was transiently expressed in hosts, the defense responses, including reactive oxygen species bursts, callose deposition, and expression of defense-related genes, were activated. Thus, the *A. Citrulli* growth in HrpE-pretreated hosts was suppressed. These results indicated that HrpE is essential for *A. citrulli* virulence but can also be used by hosts to help resist *A. citrulli*. Our findings provide a better understanding of the T3SS pathogenesis in *A. citrulli*, thus providing a molecular basis for biopesticide development, and facilitating the effective control of BFB.

## 1. Introduction

Bacterial fruit blotch (BFB) caused by *Acidovorax citrulli* Schaad et al., is a devastating seed-borne disease of Cucurbitaceae plants [1,2], which severely impacts cucurbit production and causes serious economic losses worldwide [3,4,5]. *Acidovorax citrulli*, a gram-negative bacterium, can be divided into two distinct groups: group I and group II [6]. Group II strains were mainly isolated from watermelon (*Citrullus lanatus* (Thunb.) Matsum. & Nakai), while group I strains were mainly isolated from various non-watermelon Cucurbitaceae plants, such as melon (*Cucumis melo* L.), cucumber (*Cucumis sativus* L.), and others [6,7,8,9]. Although some progress has been made regarding screening resistant crop varieties, no effective resistant varieties have been obtained [10,11,12]. At present, industrial production mainly relies on traditional chemical applications to limit BFB, which is not environmentally friendly and has the risk of developing chemical resistance [13,14]. In order to achieve efficient and long-term BFB prevention at a production scale, environmentally friendly methods that do no harm to biodiversity [15] need to be developed according to specific pathogenicity mechanisms [4]. Therefore, it is urgent to further elucidate the pathogenicity mechanism of *A. citrulli* and screen targets for chemical controls to facilitate effective BFB management.

During the long-term competition with pathogens, plants have evolved complicated immune systems [16,17]. The primary immune system of plants is initiated by conserved pathogen-associated molecular patterns (PAMPs), called PAMPs-triggered immunity (PTI). To resist PTI, pathogens secrete effector proteins into host cells, causing plant susceptibility. However, plants activate effector-triggered immunity (ETI) via a class of nucleotide-binding and leucine-rich repeat receptor proteins (NLR) [18,19]. PTI and ETI are not independent and promote each other. [20,21,22]. Revealing the molecular basis of plant-microbe interactions is the foundation to realizing efficient prevention in production [22].

The type three secretion system (T3SS) is essential and irreplaceable for the infectious cycle of many pathogenic bacteria. Any damage to the structure or function of T3SS directly affects the pathogenicity of plant pathogenic bacteria [23,24,25]. T3SS of phytopathogenic bacterium is encoded by the *hrp* (hypersensitive response and pathogenicity) gene cluster [24], and can be divided into two types according to the differences in structure, sequence, and regulation system. The first group (group I) consists of bacteria such as *Pseudomonas* (Migula) spp. and *Erwinia* (Winslow et al.) spp., etc., while the second group (group II) consists of *Acidovorax* (Willems et al.) spp., *Xanthomonas* (Dowson) spp., and *Ralstonia* (Yabuuchi et al.) spp., etc. T3SS from the same group have high similarity [25].

In plant pathogens, the structural gene *hrpE* is indispensable to T3SS, and encodes the acicular structure of the Hrp pilus [26]. Virulence factors such as effector proteins are directly injected into host cells via the Hrp pilus to facilitate the infection and colonization of the pathogen [27]. The absence of *hrpE* results in the loss of pathogenicity of plant pathogenic bacteria [27,28,29]. For example, in *Xanthomonas*, the deletion of *hrpE* leads to loss of pathogenicity in hosts and hypersensitive response (HR) in non-hosts [29]. Previous work showed that the *hrpE* mutant loses the pilus at the bacterial surface and thus cannot adhere to the surface of the host cell, losing the secretion function and the ability to interact with hosts [25,27,30].

Two recent studies have shown that HrpE may play more than a virulence role during pathogen-host interactions. HrpE was found to be recognized by host cells and induce host immune response and could therefore help resist the invasion of pathogenic bacteria to some extent [30,31]. HrpE even enhanced leaf photosynthesis efficiency in the host [31,32]. Based on the results above, we speculate that HrpE may serve as a PAMP, further, a harpin that can be recognized by host plants and induce host PTI. This point has been hypothesized previously [30,33], but not yet confirmed. Harpins are glycine-rich proteins in pathogens that induce hypersensitive cell death, elicit PTI, develop systemic acquired resistance (SAR) in various host plants, and are considered a potential component of PAMPs [31,34,35,36,37,38].

In recent years, research has progressed on the T3SS pathogenesis of *Pseudomonas* and *Xanthomonas*, but research on the T3SS pathogenesis of *A. citrulli* remains limited. It has been shown that *hrpG* and *hrpX* of *A. citrulli* are key regulators in T3SS, which regulate the downstream expression of *hrp* genes [39]. Furthermore, the deletion of regulators or structural genes such as *hrpG*, *hrpX*, *hrcN*, and *hrcQ* significantly reduces or eliminates *A. citrulli* virulence [39,40,41]. Additionally, type three effectors (T3Es) promote *A. citrulli* infection by inhibiting the immune responses of host plants [42,43,44,45]. To date, several T3Es have been characterized [42,43,44,45,46]. The model plant *Nicotiana benthamiana* Domin can be used to study *A. citrulli* [46], which has furthered research on T3SS in *A. citrulli*. However, *A. citrulli* T3SS is not fully characterized and requires further investigation.

The function of HrpE in *A. citrulli* has not been reported. To analyze the virulence role of *A. citrulli* HrpE, pathogenicity on host watermelon and *N. benthamiana*, HR on non-host tobacco, in vitro and in vivo growth, biofilm formation, and swimming motility and twitching motility of wildtype, mutant, and complementary strains were measured. Moreover, to reveal the avirulence function of HrpE and determine the potential use of HrpE to prevent *A. citrulli* infection, reactive oxygen species (ROS) production, callose deposition, and expression level of defense-related genes in host cells were measured. The findings of this study serve to further reveal the pathogenic mechanisms of *A. citrulli* T3SS and lay a foundation for effective control of BFB.

## 2. Results

### 2.1. Confirmation of the Mutant and Complementary Strains

*Acidovorax citrulli* group II strain Aac5 was used to construct the *hrpE*-deleted mutant strain and complementary strain. The open reading frame (ORF) of *hrpE* from Aac5 was found to be 100% identical to the sequence of *Aave_0464* from *A. citrulli* strain AAC00-1 (GenBank accession number CP000512.1) (Figure S1). The successful construction of the *Aave_0464* mutant Δ*hrpE* in Aac5 was confirmed by polymerase chain reaction (PCR) amplification and a 463 bp fragment was obtained using *hrpE*-UD-F/R primers (Table S1). The complementary strain ∆*hrpE*-comp showed resistance to kanamycin and yielded a 700 bp fragment when amplified with primers Kan^r^-F/R, indicating that the complementation vector pBBR-*hrpE* was successfully transferred into ∆*hrpE*. The presence of pBBR-*hrpE* in Δ*hrpE*-comp was further confirmed with primers *hrpE*-JC-F/R (Table S1) yielding a 734 bp fragment. In order to eliminate the impact of the plasmid, the empty vector was transferred into the wild-type Aac5 strain and the Δ*hrpE* strain simultaneously. Primer pair Kan^r^-F/R (Table S1) was used to confirm the successful construction of strain Aac5-pBBR and Δ*hrpE*-pBBR.

### 2.2. HrpE Is Essential for A. citrulli Pathogenicity

In order to reveal the role of *hrpE* in *A. citrulli* pathogenicity, we carried out seed-to-seedling transmission assays, watermelon spray-inoculation assays, and *Nicotiana benthamiana* inoculation assays.

**Figure 1:**
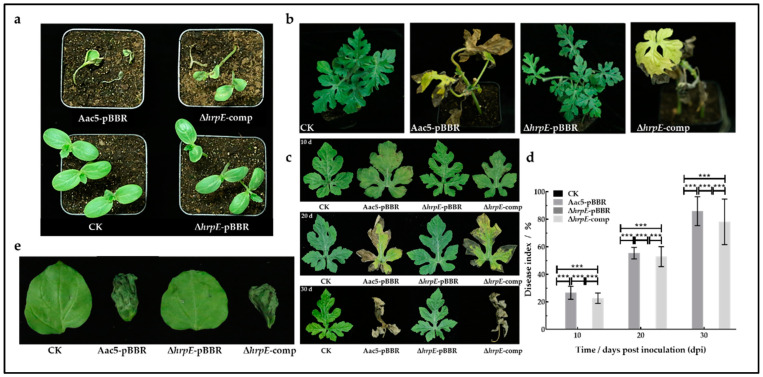
Role of *hrpE* in *A. citrulli* pathogenicity. (**a**) Watermelon seeds were soaked in Aac5-pBBR, Δ*hrpE*-pBBR, Δ*hrpE*-comp suspensions, and sterilized water (CK) respectively for 4 h, sown, and cultivated in plastic pots. Pictures were taken at 14 dpi. (**b**) Watermelon seedlings spray-inoculated with Aac5-pBBR, Δ*hrpE*-pBBR, Δ*hrpE*-comp suspensions, and sterilized water (CK) at 30 dpi. (**c**) Watermelon leaves at 10, 20, and 30 dpi inoculated with Aac5-pBBR, Δ*hrpE*-pBBR, Δ*hrpE*-comp suspensions, and sterilized water (CK). (**d**) The average disease indices (DI) of spray-inoculated watermelon seedlings at 10, 20, and 30 dpi. Asterisks represent significant differences at a given time point (two-way analysis of variance and Tukey–Kramer’s honestly significant difference test, *p* < 0.05). (**e**) *Nicotiana benthamiana* leaves at 72 h post inoculation inoculated with *A. citrulli* suspensions and sterilized water (CK).

In the seed-to-seedling transmission assays, seedlings sprouted from seeds soaked in Aac5-pBBR and Δ*hrpE*-comp suspensions showed severe BFB symptoms while the Δ*hrpE*-pBBR treated seedlings showed no symptoms and were indistinguishable from those treated with sterilized water (negative control) (Figure 1a). This indicated that the absence of *hrpE* prevents *A. citrulli* from adhering to seeds and transferring to seedlings.

In the watermelon spray-inoculation assays, for wild-type and complementary strain treated plants, dark brown spots appeared on some leaves at 10 days post inoculation (dpi). Most leaves showed BFB symptoms at 20 dpi, and almost all leaves showed severe necrosis at 30 dpi. No disease symptoms were present on mutant treated plants and negative control plants (Figure 1b,c). The average disease indices (DI) of plants inoculated with wild-type strain were 26.67 (10 dpi), 55.41 (20 dpi), and 85.97 (30 dpi). The average DIs of plants inoculated with complementary strain were 22.71 (10 dpi), 52.89 (20 dpi), and 78.18 (30 dpi), and were statistically similar to those of the wild-type strain. The DIs of the mutant and mock-treated plants (CK) remained 0 during the experiment, which were significantly lower than those of the wild-type and complementary strains (Figure 1d).

*N. benthamiana* leaves inoculated with Aac5-pBBR and Δ*hrpE*-comp suspensions showed water-soaked necrotic lesions at 72 h post inoculation (hpi), while the Δ*hrpE*-pBBR suspensions and sterilized water-treated leaves had no symptoms (Figure 1e). These results suggest that the *hrpE* mutant strain lacks pathogenicity on host watermelon and *N. benthamiana* plants.

All results above indicated that *hrpE* is essential for the pathogenicity of *A. citrulli*, and the absence of *hrpE* made *A. citrulli* lose the seed-to-seedling transmission and disease-causing capabilities in hosts.

### 2.3. A. citrulli HrpE Is Essential for HR on Non-Host Plants

**Figure 2:**
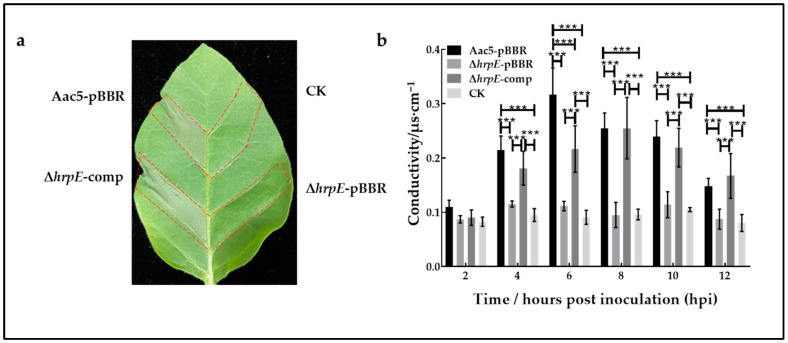
Effects of *hrpE* in inducing hypersensitive responses in non-host tobacco. (**a**) *Nicotiana tabacum* var. Samsun leaves inoculated with Aac5-pBBR, Δ*hrpE*-pBBR, Δ*hrpE*-comp strains, and sterilized water (CK) at 48 h post inoculation. (**b**) The electrolyte leakage induced by Aac5-pBBR, Δ*hrpE*-pBBR, Δ*hrpE*-comp strains, and sterilized water (CK) in *N. tabacum* var. Samsun leaves tissue. Measurements are the averages (*n* = 18) of the conductivity of the bathing water (µs/cm) ± standard deviation. Asterisks at each time point indicate significant differences (two-way analysis of variance and Tukey–Kramer’s honestly significant difference test, *p* < 0.05).

To determine the role of *hrpE* in *A. citrulli* virulence, HR induced by strains on non-host tobacco was tested. Aac5-pBBR, Δ*hrpE*-pBBR, Δ*hrpE*-comp strains and sterilized water were injected into *Nicotiana tabacum* var. Samsun NN leaves and cultivated for 48 h. The results showed that Δ*hrpE*-pBBR could not cause HR on non-host tobacco, while Aac5-pBBR and Δ*hrpE*-comp could cause HR (Figure 2a).

Quantitatively, the electrolyte leakage in leaves inoculated with Δ*hrpE*-pBBR was almost identical to the negative control indicating that the cell integrity of tobacco leaves was nearly intact. Electrolyte leakage in leaves inoculated with the wild-type strain increased quickly and reached a peak at 6 hpi, and gradually decreased, indicating that the tobacco cells became fully damaged. Electrolyte leakage in leaves inoculated with the complementary strain peaked at 8 to 10 hpi and then decreased, showing that the tobacco cells also became fully damaged, but the rate of cell damage induced by the complementary strain was slower than that of the wild-type strain (Figure 2b).

Overall, the loss of HR on non-host tobaccos confirms the importance of HrpE in *A. citrulli* pathogenesis.

### 2.4. HrpE Reduces the Growth Ability of A. citrulli In Vitro and In Vivo

The growth ability of bacteria is positively correlated with its pathogenic function [47]. The effect of *hrpE* on growth ability was measured both in vitro and in vivo.

**Figure 3:**
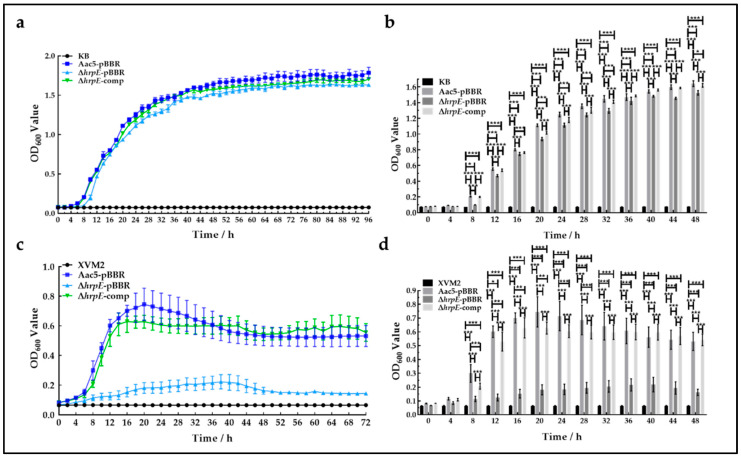
Effects of *hrpE* on in vitro growth. (**a**) Growth curve of Aac5-pBBR, Δ*hrpE*-pBBR, and Δ*hrpE*-comp strains in KB broth, using KB as the negative control (CK). (**b**) Absorbance of Aac5-pBBR, Δ*hrpE*-pBBR, and Δ*hrpE*-comp strains in KB broth at 0~48 h. Asterisks at each time point indicate significant differences (two-way analysis of variance (ANOVA) and Tukey-Kramer’s honestly significant difference test), as “*” represent *p* < 0.05, and “***” represent *p* < 0.001. (**c**). Growth curve of Aac5-pBBR, Δ*hrpE*-pBBR, and Δ*hrpE*-comp strains in XVM2 broth, using XVM2 as the negative control (CK). (**d**) Absorbance of Aac5-pBBR, Δ*hrpE*-pBBR, and Δ*hrpE*-comp strains in XVM2 broth at 0~48 h. Asterisks at each time point indicate significant differences (two-way ANOVA and Tukey–Kramer’s honestly significant difference test), as “*” represent *p* < 0.05, “**” represent *p* < 0.01, and “***” represent *p* < 0.001.

In vitro, we measured the growth rates of Aac5-pBBR, Δ*hrpE*-pBBR, and Δ*hrpE*-comp strains in nutrient-rich King’s B (KB) broth [39] and nutrient-poor XVM2 broth [48], with the medium broths as the negative controls. In KB broth, the mutant strain entered the logarithmic growth phase later compared with the wild-type strain. The growth rate (indicated by the absorbance) of the mutant strain was significantly lower than that of wild-type strain after the first 4 h (Figure 3a,b). In XVM2 broth, the growth rate of the mutant strain was significantly lower than that of the wild-type strain, diverging from each other at 2 h and staying apart for the length of the experiment, with the peak of the wild-type strain almost four times that of the mutant strain (Figure 3c,d).

These results revealed that the absence of *hrpE* reduces the in vitro growth capability of *A. citrulli*, and this reduction differential was greater when nutritional conditions were poor.

**Figure 4:**
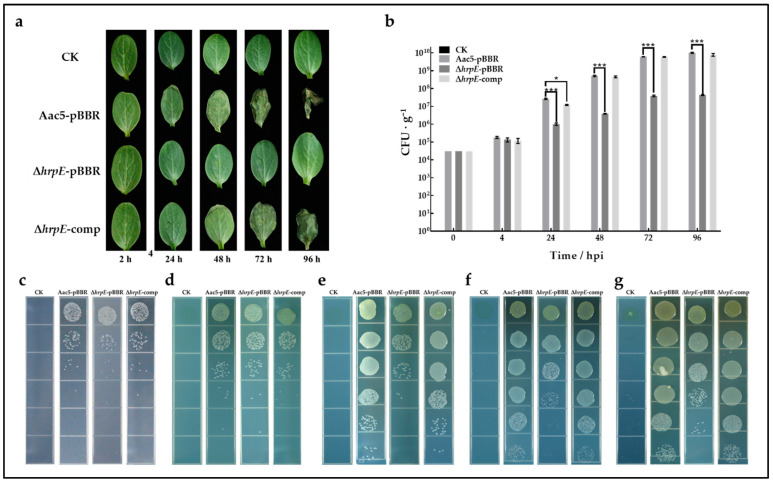
Effect of *hrpE* on colonizing watermelon cotyledons. (**a**) Images of watermelon cotyledons inoculated with Aac5-pBBR, Δ*hrpE*-pBBR, Δ*hrpE*-comp strains, and sterilized water (CK) at 4, 24, 48, 72, and 96 h post inoculation (hpi). (**b**) Bacterial population levels in watermelon cotyledon leaf disks inoculated with tested strains at 2, 24, 48, 72, and 96 hpi. Bars indicate the standard errors of the means of three replicated experiments, each consisting of six cotyledon disks per strain per time point. Asterisks at each time point represent significant differences compared with the wild-type Aac5-pBBR strain (two-way analysis of variance and Tukey–Kramer’s honestly significant difference test), as “*” represent *p* < 0.05, and “***” represent *p* < 0.001. (**c**–**g**) *A. citrulli* colonies on KB plates isolated from leaf disks of watermelon cotyledons inoculated with tested strains at 4 (**c**), 24 (**d**), 48 (**e**), 72 (**f**), and 96 (**g**) hpi.

In vivo, watermelon cotyledons were inoculated with Aac5-pBBR, Δ*hrpE*-pBBR, Δ*hrpE*-comp strains, and sterilized water, and sampled at 4, 24, 48, 72, and 96 hpi. Cotyledons treated with Aac5-pBBR and Δ*hrpE*-comp strains showed BFB symptoms gradually, while cotyledons treated with Δ*hrpE*-pBBR strain and sterilized water showed no symptoms (Figure 4a). The population levels of tested strains in cotyledons were detected quantitatively by counting the colonies on KB plates. There was no significant difference among the population levels of tested strains during the first 4 h. The population levels of wild-type strains and complementary strains were similar statistically at all time points and were significantly larger than those of the *hrpE* mutant strain at 24~96 hpi (Figure 4b–g). These results revealed that the absence of *hrpE* reduced the colonization ability of *A. citrulli* in the host watermelon.

To sum up, the absence of *hrpE* reduced the growth ability of *A. citrulli* both in vitro and in vivo.

### 2.5. Deletion of hrpE Enhanced the Biofilm Formation Ability of A. citrulli

**Figure 5:**
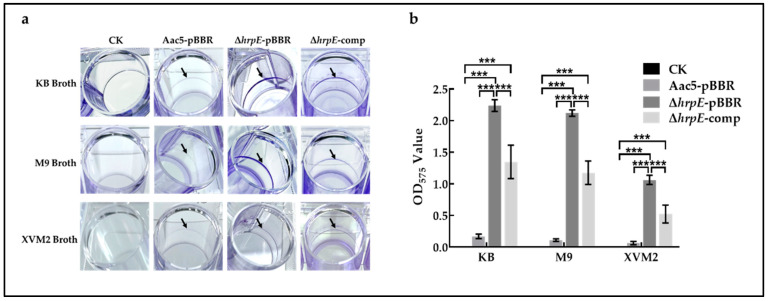
Effect of *hrpE* on biofilm formation in medium broth. (**a**) Aac5-pBBR, Δ*hrpE*-pBBR, and Δ*hrpE*-comp strains formed visible biofilm rings on the inner wall of culture plates in different medium broths, with the media as the negative control (CK). (**b**) Biofilm formed by tested strains was dissolved in 95% ethanol, and the absorbance of the solution was tested at OD_575_. The bars represent standard errors of the means, and asterisks represent significant differences (two-way analysis of variance and Tukey–Kramer’s honestly significant difference test, *p* < 0.05).

Biofilm of phytopathogenic bacteria is conducive to the adaptation of pathogens to the environment and helps infect host plants [49]. To determine the role of *hrpE* in *A. citrulli* biofilm formation, the tested strains were cultivated in three different nutrient media: KB (nutrient-rich medium), M9 (nutrient-poor medium), and XVM2 (nutrient-poorer medium), with the media as the negative control (CK). The Δ*hrpE*-pBBR and Δ*hrpE*-comp strains formed visible biofilm rings on the inner walls of polystyrene cell culture plates. The wild-type strain formed an almost invisible biofilm ring in all three media broths (Figure 5a). Furthermore, in the quantitative assay, the absorbance of the mutant strain and complementary strain in all three media were significantly higher than that of the wild-type strain (Figure 5b). These indicated that the deletion of *hrpE* significantly enhances the biofilm formation of *A. citrulli*, and the degree of the enhancement was medium-dependent.

### 2.6. Deletion of hrpE Reduced the Swimming Motility and Twitching Motility of A. citrulli

**Figure 6:**
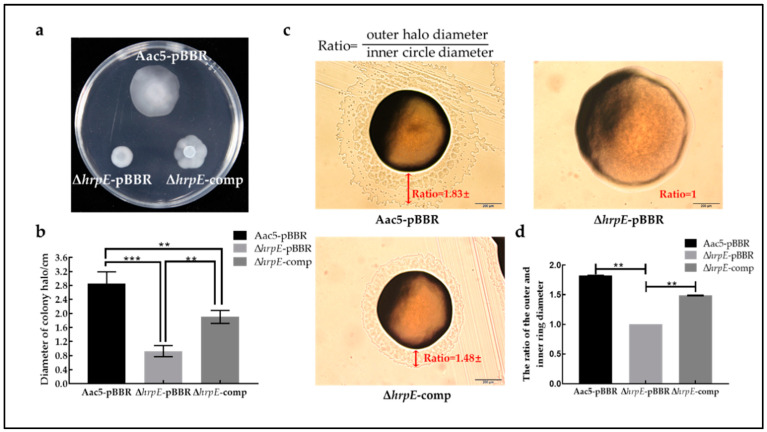
Effect of *hrpE* on swimming motility and twitching motility of *A. citrulli*. (**a**) The swimming motility of tested strains showed as white halos on the 0.3% agar medium when cultivated for 72 h. (**b**) The average halo diameter of Aac5-pBBR, Δ*hrpE*-pBBR, and Δ*hrpE*-comp strains for ten replicates. The bars represent standard errors of the means, and asterisks indicate significant differences (One-way analysis of variance (ANOVA) and Tukey–Kramer’s honestly significant difference test, *p* < 0.05). (**c**) Twitching motility of Aac5-pBBR, Δ*hrpE*-pBBR, and Δ*hrpE*-comp strains incubated on KB plates for 48 h. Ratio means the ratio of the outer halo diameter and inner circle diameter. (**d**) The mean ratio of outer halo diameter to inner circle diameter of tested strains for ten replicates (mean ± standard error); asterisks indicate significant statistical differences (One-way ANOVA and Tukey–Kramer’s honestly significant difference test), as “**” represent *p* < 0.01, and “***” represent *p* < 0.001.

Motility can promote the adhesion and invasion of bacteria to host cells, which is closely related to biofilm formation and bacterial virulence [50]. The swimming motility and twitching motility of Aac5-pBBR, Δ*hrpE*-pBBR, and Δ*hrpE*-comp strains were tested. The swimming motility of Δ*hrpE*-pBBR (mean diameter = 0.80 cm), and Δ*hrpE*-comp strains (mean diameter = 1.85 cm), was significantly lower than that of Aac5-pBBR (mean diameter = 2.81 cm), and the swimming motility of Δ*hrpE*-comp was partially restored to the wild-type level (Figure 6a,b). Regarding twitching motility assays, there was no halo outer the Δ*hrpE*-pBBR strain, which means the twitching motility of the mutant strain was lost. Both Aac5-pBBR and Δ*hrpE*-comp strains had outer halos, and the mean ratio of outer halo diameter and inner circle diameter of Δ*hrpE*-comp (1.48) was significantly smaller than that of the Aac5-pBBR strain (1.83) (Figure 6c,d).

Results of swimming motility and twitching motility assays revealed that HrpE was positively correlated with motility in *A. citrulli*.

### 2.7. Pre-Treatment with HrpE Helps Host Plants Resist A. citrulli Infection

To confirm whether HrpE could help hosts resist the infection of *A. citrulli*, watermelon cotyledons and *N. benthamiana* leaves were pre-treated with transiently expressed HrpE protein. Leaves were injected with Aac5 suspensions 48 h after HrpE treatment.

**Figure 7:**
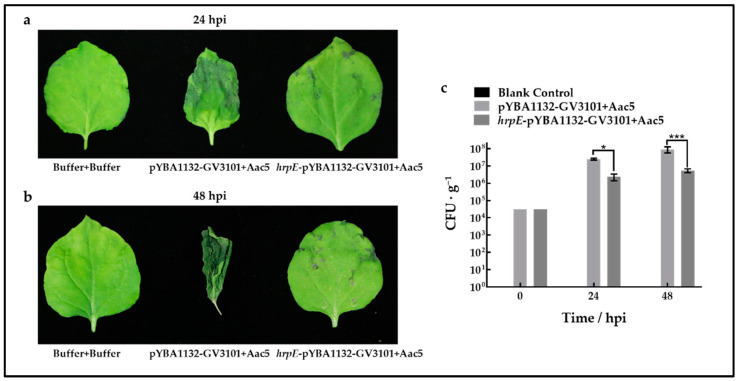
Effects of HrpE on resistance to *Acidovorax citrulli* infection in *Nicotiana benthamiana*. *N. benthamiana* leaves were pre-injected with *Agrobacterium tumefaciens* pYBA1132-GV3101 and *hrpE*-pYBA1132-GV3101 for 48 h, using the buffer as the negative control. Images were taken at 24 hpi (**a**) and 48 hpi (**b**) with Aac5, with the buffer serving as the negative control. (**c**) Aac5 population levels from *N. benthamiana* leaf disks from six replicates treated with pYBA1132-GV3101+Aac5 and *hrpE*-pYBA1132-GV3101+Aac5, using the buffer + buffer treatment as the negative control. The bars represent standard errors of the means from three experimental treatments each consisting of six cotyledon disks. Asterisks indicate significant statistical differences (two-way ANOVA), as “*” represent *p* < 0.05, and “***” represent *p* < 0.001.

The *N. benthamiana* leaves pre-treated with HrpE showed few BFB symptoms after treatment with Aac5, while leaves without HrpE-treatment showed progressively more severe symptoms (Figure 7a,b). The population levels of Aac5 in HrpE-pretreated *N. benthamiana* leaves were significantly less abundant than those without HrpE-treatment at 24 and 48 hpi (Figure 7c).

**Figure 8:**
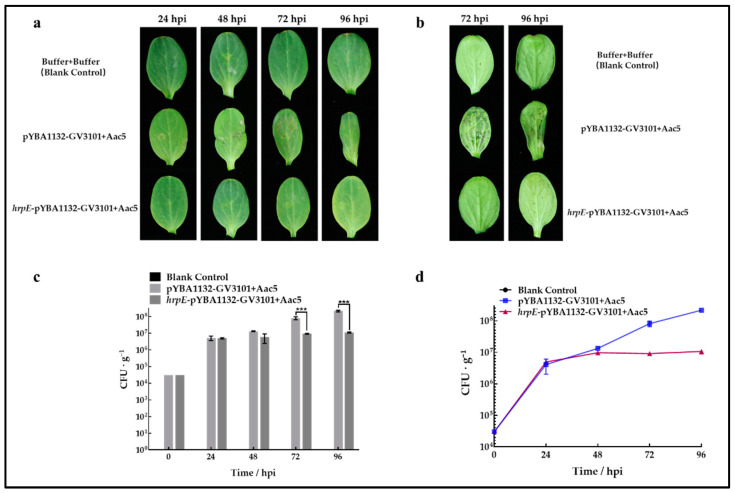
Effect of HrpE on resistance to *A. citrulli* infection in watermelon. (**a**) Watermelon cotyledons were pre-injected with *Agrobacterium tumefaciens* pYBA1132-GV3101 and *hrpE*-pYBA1132-GV3101, then injected with Aac5 suspensions 48 h later, using 10 mM MgCl_2_ solution as the negative control. Samples were observed and photographed at 24, 48, 72, and 96 h after Aac5 inoculation. (**b**) Symptoms on the underside of cotyledons were observed and photographed at 72 and 96 h after Aac5 inoculation. (**c**) The colony counts of Aac5 population levels from watermelon cotyledon disks treated with pYBA1132-GV3101+Aac5 and *hrpE*-pYBA1132-GV3101+Aac5. A solution of 10 mM MgCl_2_ was used as the negative control. The bars represent standard errors of the means from three experimental treatments, each consisting of six cotyledon disks. The asterisks indicate significant differences compared to the positive control pYBA1132-GV3101+Aac5 (two-way analysis of variance, *p* < 0.05). (**d**) Growth curves of Aac5 in HrpE-pretreated and non-HrpE-treated cotyledons. The bars represent standard errors of the means from three experimental treatments each consisting of six cotyledon disks.

These findings were consistent with assays on watermelon cotyledons. At 48 to 96 h after injection with Aac5, water-soaked lesions appeared gradually on non-HrpE-treated cotyledons, while HrpE-pretreated cotyledons did not present mild lesions until 96 h (Figure 8a). Symptoms were more apparent on the abaxial side of the cotyledons than on the adaxial side (Figure 8b). The population levels of Aac5 in HrpE-pretreated watermelon cotyledons mostly remained at 10^6^ CFU·g^−1^, while those of non-HrpE-treated cotyledons increased continually to 10^8^ CFU·g^−1^. There were significant differences at 72 hpi and 96 hpi (Figure 8c,d).

These results confirmed the hypothesis that pre-treatment with HrpE could suppress the growth of *A. citrulli* in host plants, which indicates that the HrpE protein could be used by hosts to resist *A. citrulli* infection.

### 2.8. HrpE Localized at Cytomembrane and Nuclear in Hosts

**Figure 9:**
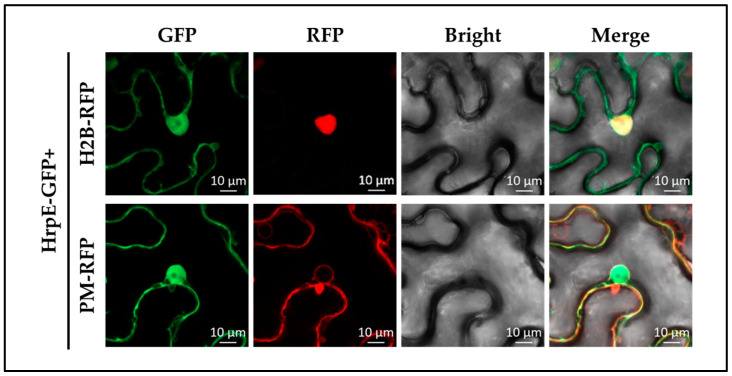
Subcellular localization of HrpE in *Nicotiana benthamiana*. *N. benthamiana* leaves syringe injected with *Agrobacterium tumefaciens* pYBA1132-GV3101+PM, pYBA1132-GV3101+H2B-RFP, *hrpE*-pYBA1132-GV3101+PM, and *hrpE*-pYBA1132-GV3101+H2B-RFP suspensions at OD_600nm_ = 0.3. Suspensions were pretreated in darkness for 3 h before injection. PM represents the plasma membrane marker carrying RFP protein, which was localized at entire cell membranes; H2B-RFP represents the cell nucleus marker carrying RFP protein localized at nuclear. Images (15 per treatment) were taken with confocal microscopy at 60× magnification. The scale bar represents 10 μm.

To investigate the role of HrpE in host interaction, the subcellular localization of HrpE was visualized by co-expressing eGFP (enhanced green fluorescent protein) in *N. benthamiana*. Fluorescence of HrpE-eGFP was observed in the cytomembrane, nuclear membranes, and skeleton but not nucleolus of *N. benthamiana* cells (Figure 9). The plasma membrane marker PM carrying red fluorescent protein (RFP) was localized throughout the cytomembrane while the cell nucleus marker H2B-RFP carrying an RFP tag was only localized in the entire nucleus. The superposition of green fluorescence (emitted by HrpE-eGFP) and red fluorescence (emitted by the RFP marker) revealed that the HrpE indeed localizes at cytomembrane and nuclear (not nucleolus).

### 2.9. HrpE Induces ROS Burst in Hosts

**Figure 10:**
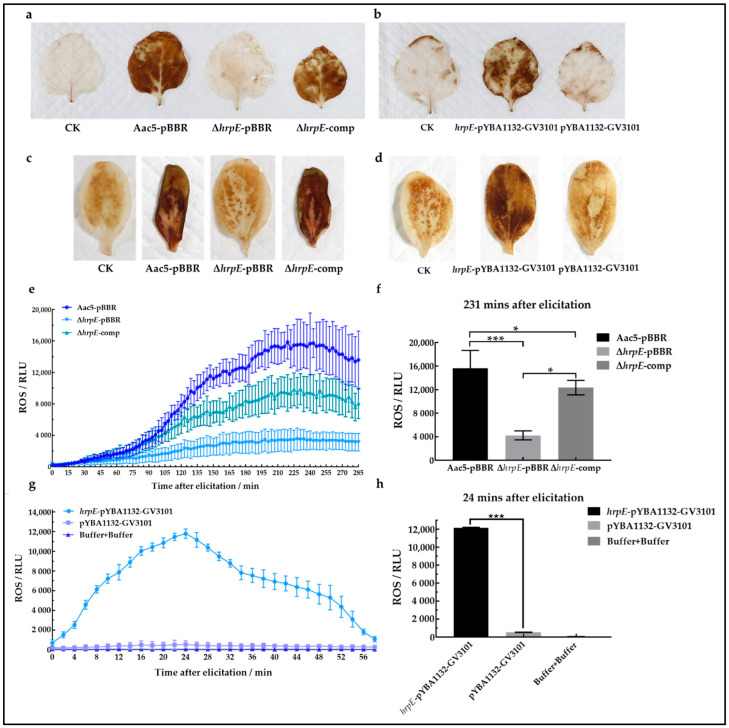
HrpE induces ROS burst in hosts. *Nicotiana benthamiana* leaves (**a**) and watermelon cotyledons (**c**) were syringe-injected with Aac5-pBBR, Δ*hrpE*-pBBR, and Δ*hrpE*-comp suspensions at OD_600nm_ = 0.3, using the buffer as the negative control (CK). At 48 h post inoculation (hpi), leaves were collected, stained with DAB (3′3-diaminobenzidine), boiled in 95% ethanol in a water bath at 99.99 °C until completely discolored, and photographed. *N. benthamiana* leaves (**b**) and watermelon cotyledons (**d**) were injected with *hrpE*-pYBA1132-GV3101 and pYBA1132-GV3101 suspensions at OD_600nm_ = 0.3, using the same buffer as the negative control (CK). Similarly, leaves were collected at 48 hpi, stained with DAB, discolored, and photographed. *N. benthamiana* leaves were syringe-injected with Aac5-pBBR, Δ*hrpE*-pBBR, and Δ*hrpE*-comp suspensions at OD_600nm_ = 0.3 (**e**) or injected with *hrpE*-pYBA1132-GV3101 and pYBA1132-GV3101 suspensions at OD_600nm_ = 0.3 (**g**), using the buffer as the negative control (CK). Leaf disks (*n* = 36) from each treatment were collected and placed in a 96-well microplate with luminol solution. The luminescence intensity of each well was measured, and the means of 108 replicates for each treatment were plotted with error bars indicating the standard error. Maximum values in (**e**) (231 min after elicitation) (**f**,**g**) (24 min after elicitation) (**h**) were selected and plotted. The bars represent standard errors of the means, and asterisks indicate significant differences between treatments (one-way analysis of variance), as “*” represent *p* < 0.05 and “***” represent *p* < 0.001.

To further explore the host responses to HrpE, host ROS production was measured, which is one of the markers of host immunity response [51]. 3′3-diaminobenzidine (DAB) staining showed that both the *N. benthamiana* and watermelon leaves syringe-injected with Aac5-pBBR and Δ*hrpE*-comp suspensions were almost entirely covered with brown precipitate, while leaves injected with Δ*hrpE*-pBBR barely produced any brown precipitate (Figure 10a,c). This suggested that the absence of *hrpE* in the pathogen may reduce host ROS production.

Similarly, leaves treated with *hrpE*-pYBA1132-GV3101 visibly produced more brown precipitates than pYBA1132-GV3101 treatment (Figure 10b,d). To quantify these observed differences, the luminescence emitted by pre-treated *N. benthamiana* leaf disks was measured. The results showed that in the absence of *hrpE*, ROS production induced by *A. citrulli* was significantly reduced. Similarly, the presence of the HrpE protein could significantly enhance ROS production, suggesting that HrpE triggers ROS bursts in host cells (Figure 10e–h). These findings further confirmed that HrpE could induce ROS bursts in host cells and plays an important role in the pathogen-host interactions.

### 2.10. HrpE Induced Callose Deposition in Hosts

**Figure 11:**
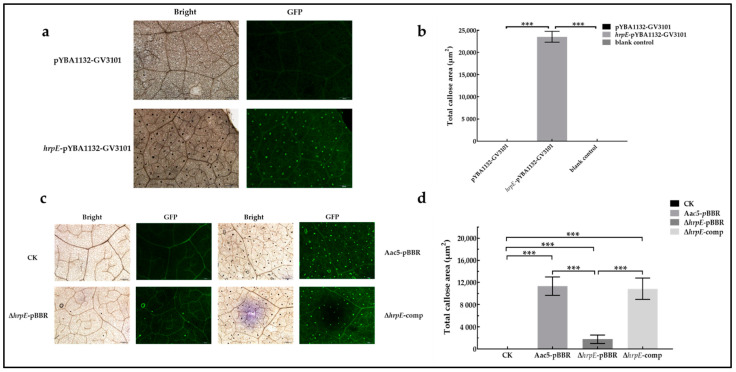
HrpE induced callose deposition in host cells. (**a**) *Nicotiana benthamiana* leaves were treated with *Agrobacterium tumefaciens hrpE*-pYBA1132-GV3101 and pYBA1132-GV3101 suspensions at OD_600nm_ = 0.3. The buffer solution was used as the blank control. Leave disks (*n* = 30/treatment) were collected at 20 hpi, discolored and stained with aniline blue solution (0.04% aniline blue in 150 mM K_2_HPO_4_ solution), observed under fluorescence microscopy, and photographed. (**b**) The total callose area produced in hosts treated with pYBA1132-GV3101 and *hrpE*-pYBA1132-GV3101 was measured using Image J, and values are the means calculated from 30 different photographs. Error bars represent the standard error. Asterisks indicate significant differences compared to the pYBA1132-GV3101 treatment (one-way ANOVA, *p* < 0.05). (**c**) *N. benthamiana* leaves were treated with Aac5-pBBR, Δ*hrpE*-pBBR, and Δ*hrpE*-comp suspensions at OD_600nm_ = 0.3. The buffer solution was used as the blank control. Leaf disks were collected at 20 hpi, discolored and stained with aniline blue, and observed with fluorescence microscopy (15 photographs/treatment). (**d**) The total area of callose produced in hosts injected with Aac5-pBBR, Δ*hrpE*-pBBR, and Δ*hrpE*-comp suspensions was measured using Image J, and values are the mean calculated from 15 different photographs. The buffer solution was used as the blank control (CK). Error bars represent the standard error and asterisks indicate significant differences compared to wild type (one-way ANOVA, *p* < 0.05).

Callose deposition is a marker of PTI responses in host plants [21,52]. The amount of callose produced in watermelon cells cannot be quantified due to the thickness of watermelon cotyledons. Therefore, this experiment was performed with *N. benthamiana*. Leaves treated with the *hrpE*-pYBA1132-GV3101 suspension produced large quantities of callose, which was stained brown with aniline blue and emitted green fluorescence (Figure 11a). Leaves treated with the pYBA1132-GV3101 suspension did not produce any callose (Figure 11a). The total callose area between treatments was significantly different (Figure 11b), indicating that HrpE induces the production and deposition of callose in host cells.

The deposition of callose in leaves injected with Aac5-pBBR was significantly greater than that of Δ*hrpE*-pBBR injected leaves, and similar to that of Δ*hrpE*-comp injected leaves (Figure 11c,d). The absence of HrpE reduced the production and deposition of callose in the host cell.

The results of the above two experiments were correlated, revealing that HrpE can induce callose deposition in host cells.

### 2.11. Expression of PTI-Marker Genes Induced by HrpE in Tobacco

**Figure 12:**
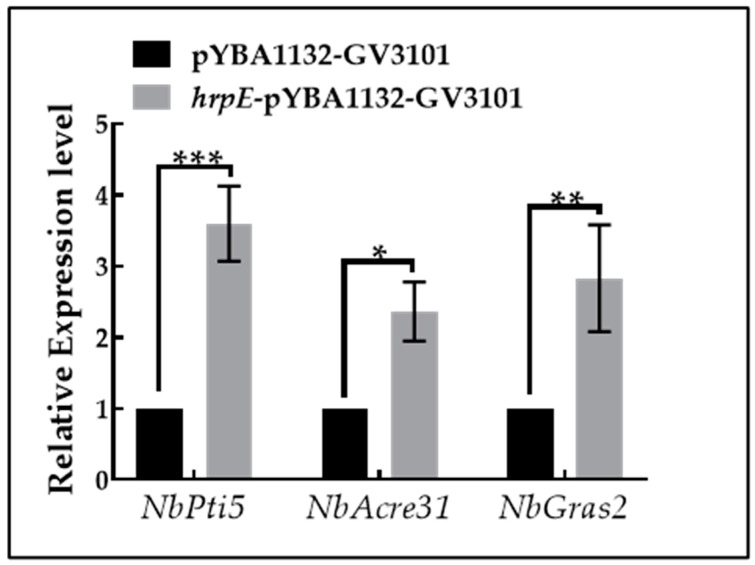
The relative expression levels of PTI-marker genes in inoculated *Nicotiana benthamiana* leaves. *Agrobacterium tumefaciens hrpE*-pYBA1132-GV3101 and pYBA1132-GV3101 suspensions were injected into *N. benthamiana* leaves at OD_600nm_ = 0.3 and leaves were collected from each treatment at 24 hpi. RNA was extracted from leaf tissues, and reverse transcribed into cDNA. Expression levels of *NbPti5*, *NbAcre31*, and *NbGras2* were measured by RT-qPCR. *NbEF1α* was used as a reference gene. The 2^−ΔΔCT^ method was used to calculate the relative expression of genes. Mean values were calculated from three replicates, and error bars indicate the standard error. Asterisks represent significant differences between treatments (*t*-test), as “*” represent *p* < 0.05, “**” represent *p* < 0.01, and “***” represent *p* < 0.001.

In addition to ROS bursts and callose deposition, the host PTI response also includes the expression of defense-related genes [20,21,22]. Therefore, the relative expression levels of PTI-marker genes including *NbPti5*, *NbAcre31*, and *NbGras2* [21] in *N. benthamiana* leaves were measured at 24 hpi. The expression levels of *NbPti5*, *NbAcre31*, and *NbGras2* in *hrpE*-pYBA1132-GV3101 treated leaves were significantly higher than in pYBA1132-GV3101 treated leaves (Figure 12). This demonstrated that HrpE could induce the expression of PTI-marker genes in the host.

## 3. Discussion

The T3SS plays an essential role in the pathogenesis of phytopathogenic bacteria. In particular, the Hrp pilus encoded by *hrpE* is an important component of T3SS [27]. Previous research showed that HrpE serves as a conduit to transfer virulence factors such as effectors into the host plant cell cytosol, facilitating infection of host plants [27,33,53,54,55,56,57]. Although previously considered simply pathogenic, recently, two studies have shown that HrpE may play additional roles in pathogen-host interactions [30,31]. We speculate that a broader function of HrpE may also exist in *A. citrulli*. Due to the relatively limited research on *A. citrulli*, and difficulty in genetic manipulation due to its high GC content, little is known about the *A. citrulli* T3SS. In this study, functional analysis of HrpE using *A. citrulli* group II strain Aac5 as a model reveals the functions of this important protein from both disease-causing and disease-resistant perspectives.

To analyze the function of HrpE in T3SS pathogenesis, a series of virulence assays were carried out. The results showed that the Δ*hrpE* strain lost virulence in hosts and non-hosts, which is consistent with previous studies [27,30,31,53,54]. The virulence of T3SS is mainly carried out by T3Es [58,59]. Previously, T3Es have been shown to assist pathogen infection and colonization of the host by regulating physiological activities and inhibiting host defense responses [58,59,60]. The domain predictor SMART (http://smart.embl-heidelberg.de, accessed on 15 January 2019) showed HrpE contained a FliH domain from the 95th to 255th amino acid, a region also found in the flagellar assembly protein FliH, which is involved in flagellum-specific export processes. The FliH domain might suggest the effector secretion function of HrpE. Therefore, it is likely that the lack of virulence in the mutant strain may be attributed to the loss of the transfer channel encoded by *hrpE* for effectors. In short, without HrpE, T3Es cannot be transferred into host cells and thus the bacterial pathogen loses virulence mechanisms. This is consistent with previous research on *Xanthomonas* and *Pseudomonas* [27,28,53,54,55,56,57].

Growth ability is one of the key metrics for determining the pathogenicity of pathogens [47]. In *Xanthomonas oryzae* pv. *oryzae* (Ishiyama) Swings et al. (*Xoo*), researchers observed that their loss of function HrpE mutant had a significantly lower growth rate in the liquid medium compared to the wild-type strain, however, no mechanism was investigated [31]. Consistent with previous results, the absence of *hrpE* in *A. citrulli* significantly reduced growth ability in the host and in nutrient-poor liquid medium. Entering the exponential phase was delayed for the *hrpE* mutant in the nutritious medium. This suggested that HrpE may impact the colonization capacity and the tolerance of adverse growth conditions. We speculate that the absence of *hrpE* causes loss of the Hrp pilus in bacteria, thus physically preventing pathogen interaction with the host and infusion of effectors into host cells to regulate the host’s physiological activities. This made the pathogen unable to fully utilize environmental nutrients, thus eventually manifesting as the reduction of in vivo growth ability. Here, potential functionally associated genes with HrpE in *A. citrulli* were identified using the online tool STRING (https://version11.string-db.org, accessed on 15 January 2019). HrpE might be functionally associated with the chemotaxis system modulation gene *Aave_2417*, *cheA* signal transduction histidine kinase gene *Aave_0905*, flagellar assembly gene *Aave_4429* and *Aave_4395*, and flagellar motor switch gene *Aave_4394* and *Aave_4388*. These findings indicate that HrpE might functionally link with chemotaxis and motility, as the absence of *hrpE* might affect growth ability in liquid medium via impacts on chemotaxis and motility. Without chemotaxis and motility, bacteria tend to not be able to take full advantage of nutrient-rich growth conditions rapidly. The specific underlying mechanism of these changes in growth rate requires further study.

The motility is beneficial to the survival of pathogens and facilitates infection of host plants [50]. HrpE indeed affects the motility of *A. citrulli*. The swimming motility and twitching motility were significantly reduced in the *hrpE* mutant strain compared with the wild-type strain. Similarly, the deletion of *hrpE* also reduced motility in *Xoo* [31].

Biofilm formation is an important virulence factor of pathogens, facilitating bacterial adaptation to the environment and survival, and is usually positively associated with pathogenicity [49]. In this study, the biofilm formation ability of the mutant strain was significantly increased compared with the wild-type strain, which was negatively correlated with the pathogenicity of the mutant strain. This result is consistent with Guan et al. [61] who assumed that inhibited motility promotes biofilm formation through a motility-biofilm transition. As biofilms are associated with signal transduction and quorum sensing, we also speculate that the absence of *hrpE* caused disorders of the quorum sensing system and signal transduction, thus bacterial cells multiplied out of control, and ultimately lead to this negative correlation.

Overall, *hrpE* is essential for *A. citrulli* virulence, affecting pathogenicity both directly and indirectly. The Hrp pilus encoded by *hrpE* serves as a structural component of the T3SS and participates in the pathogenesis of *A. citrulli* directly. Beyond the function of effector secretion [28,55], HrpE also regulated pathogenicity indirectly by affecting growth ability, motility, and biofilm formation.

Microbes are known to evolve with eukaryotic hosts in symbiotic, mutualistic, or parasitic relationships. The mechanisms of host–microbe interactions can provide a better understanding of the evolutionary and ecological dynamics of host and microbe. The evolution of the plant immune response is tightly linked with its pathogens [62]. PTI is the first immune defense system in plants, which is an important protective mechanism in hosts. Pattern recognition receptors (PRRs) on plant membranes can recognize PAMPs of pathogens to activate the PTI response in plants, which can inhibit the growth of bacteria [62]. Typical PTI responses include ROS production, callose deposition, and the expression of defense-related genes in hosts [63,64]. It is of great scientific significance to analyze the mechanism of plant immune formation for developing disease control methods.

To analyze the effects of HrpE in hosts, HrpE was transiently expressed in hosts and the wild-type Aac5 strain was injected into these pre-treated plants. The expression of HrpE suppressed the growth of Aac5 in host plants. These results were consistent with previous findings [30,31], suggesting that HrpE suppresses the infection of *A. citrulli* to the host. This indicates HrpE could potentially be made into a biopesticide that could be used in production.

To further determine the underlying mechanism, the subcellular localization of HrpE in host *N. benthamiana* was investigated. We found that the transiently expressed HrpE was localized at cytomembrane and nuclear, which was different from our hypothesis. We previously hypothesized that HrpE would be localized only at cytomembrane, as it was a structural component and no evidence showed that it could be secreted into the host cell. To the best of our knowledge, the subcellular localization of HrpE homologs has not been previously reported. We mainly ascribe the present results to the interaction targets of HrpE in hosts, which might bring HrpE into the nucleus. This localization may also be related to its functions of enhancing photosynthesis and promoting plant growth [30,31,32]. The specific mechanism of this phenomenon needs to be further studied and may further elucidate the role of HrpE in pathogen-host interaction.

When plants are infected by pathogens, receptor-like kinases in plants can bind to PRRs and undergo a series of phosphorylation reactions, leading to ion fluxes and ROS bursts [21,63,65]. As an early signaling molecule that activates subsequent defense responses, ROS can not only reduce microbial viability directly [17,63,66], but also participates in a variety of signaling pathways, such as callose deposition, defense gene expression, phytoalexin production, and SAR [65,66]. Callose is a polymer of β-L,3 glucan, which deposits instantaneously and reversibly on the cell walls, helping to strengthen cell walls and thus resist pathogens [52]. We found that HrpE could induce ROS burst and callose deposition, and significantly up-regulate the expression of PTI marker genes in hosts. Our results were consistent with findings in *Xoo* and *Xanthomonas campestris* pv. *campestris* (Pammel) Dowson [30,31]. Together, these results suggest that HrpE stimulates the host immune response.

Based on the results shown here, we believe that HrpE is a newly recognized PAMP, and support our previous hypothesis that HrpE serves as a harpin protein to a certain extent. When expressed in plants alone, HrpE is avirulent to plants and can activate host defense responses, which enables its potential use in the future as a biopesticide to enhance disease resistance of host plants. However, we acknowledge that there are still some limitations. In this study, the concentration of transient expression strain *hrpE*-pYBA1132-GV3101 was OD_600nm_ = 0.3, and at this concentration Aac5 infection was not fully resisted. At late infection stages, although pathogen growth was suppressed, slight symptoms still appeared on host leaves. Whether a higher inoculum concentration of the transient expression strain could prevent infection completely, and the interaction targets on hosts require further investigation.

In conclusion, *A. citrulli* HrpE can be used both by bacteria and host plants. In *A. citrulli*, *hrpE* participates in pathogenesis directly and indirectly, and its pathogenic effects are more than a mere effector secretion conduit. In host plants, HrpE stimulates the immune responses and helps suppress *A. citrulli* infection. These findings indicate that HrpE could serve as a drug target to reduce the pathogen virulence, or be turned into a biological pesticide to strengthen the plants.

## 4. Materials and Methods

### 4.1. Bacterial Strains, Plasmids, Growth Conditions, and Primer Design

*Acidovorax citrulli* wild-type strain Aac5 used in this study is a group II strain with an almost identical genomic sequence to AAC00-1 (GenBank accession number CP000512.1). The deletion mutant strain and complementary strain were constructed from Aac5. All *A. citrulli* strains were grown in King’s B (KB) or T3SS-inducing broth XVM2 [48] or on a KB plate (KB containing agar at 15 g/L) with appropriate antibiotics at 28 °C. *Agrobacterium tumefaciens* (Smith & Townsend) Conn and *Escherichia coli* (Migula) Castellani & Chalmers strains were grown in Luria-Bertani (LB) broth or on an LB plate (LB containing agar at 15 g/L) at 28 and 37 °C, respectively [67]. Antibiotics used in cultivation were ampicillin (Amp), 100 µg/mL; kanamycin (Kan), 50 µg/mL; chloramphenicol (Cm), 25 µg/mL; and rifampicin (Rif), 50 µg/mL. All primers were designed in Primer 3.0 (http://www.simgene.com/Primer3, accessed on 15 January 2019) based on the AAC00-1 genomic sequence. Bacterial strains, plasmids, and primers used in this study are listed in Tables S1 and S2, respectively.

### 4.2. Plant Materials and Culture Conditions

Watermelon (*C. lanatus*) cultivar ‘Jingxin#3′ was used in seedling assays (provided by Beijing Academy of Agriculture and Forestry Sciences, Beijing, China). Watermelon cultivar ‘Ruixin’ was used in seed assays (provided by the Institute of Vegetables and Flowers, Chinese Academy of Agricultural Sciences, Beijing, China). Watermelon plants were grown in a growth chamber with 56–65% relative humidity (RH) and a 12:12 h light: dark, 25:22 °C regime for inoculation assays. *N. benthamiana* and *N. tabacum* var. Samsun were grown in a growth chamber with 60% RH and a 16:8 h light: dark, 25:20 °C regime for transient expression and HR assays [39].

### 4.3. Construction of the hrpE Mutant and Its Complement Strain

The *hrpE* gene deletion mutant was constructed via a homologous double recombination approach [39]. Primers were designed based on the AAC00-1 sequence *Aave_0464* (*hrpE* homolog) and its flanking regions. A 337 bp fragment upstream of the *Aave_0464* open-reading frame (ORF) and a 447 bp fragment downstream of the *Aave_0464* ORF, were amplified from Aac5 by PCR. The amplified fragment was found to be 100% identical to the sequence from AAC00-1 by DNAMAN version 5.2.2 (Lynnon Biosoft, Quebec, QC, Canada). The recombinant fragment was integrated into pK18*mobsacB* [68] generating the recombinant vector pK18-*hrpE*-UD. The recombinant vector was transformed into Aac5 from DH5α [69] by triparental mating with an *E. coli* strain carrying the helper plasmid, pRK600 [70]. Transconjugants were screened on M9 screening medium plates (Na_2_HPO_4_·12 H_2_O 75.6 g, KH_2_PO_4_ 15 g, NH_4_Cl 5 g, NaCl 2.5 g, 20% sodium citrate 10 mL, 1 M MgSO_4_ 1 mL, and agar powder 15 g per liter deionized water, pH 7.0) supplemented with Amp and 10% sucrose. The primer pair *hrpE*-UD-F/R was used to verify the successful construction of the *hrpE* mutant.

The *hrpE* ORF with its upstream native promoter fragment (1383 bp) was amplified using primers *hrpE*-HB-F/R and introduced into pBBR1MCS-2 [71], to generate the complementation vector pBBR-*hrpE*. The complementation vector was then introduced into Δ*hrpE* to generate the complementary strain Δ*hrpE*-comp. Next, transconjugants were screened on KB plates supplemented with antibiotics (Amp and Kan). The *hrpE*-JC-F/R primer was used to verify that the complementary strain was successfully constructed.

Additionally, to eliminate the impact of the vector on bacterial host cells, empty vector pBBR1MCS-2 was transferred to the wild-type Aac5 strain and the Δ*hrpE* strain by triparental mating. WT-pBBR and Δ*hrpE*-pBBR strains were obtained by growing the transformants on Amp and Kan amended plates.

### 4.4. Pathogenicity Assays

To determine the role of *hrpE* in *A. citrulli* virulence, seed-to-seedling transmission assays, watermelon seedling inoculation assays, and *N. benthamiana* inoculation assays were carried out three times independently.

#### 4.4.1. Seed-to Seedling Transmission Assays

The seed-to-seedling transmission assays were performed as previously described [39], with slight modifications. Briefly, the strains Aac5-pBBR, Δ*hrpE*-pBBR, and Δ*hrpE*-comp were cultured in KB broth, centrifuged at 28 °C 5000 rpm for 10 min, and resuspended with sterilized distilled water (SDW). The concentration of resuspended strains was adjusted to 3 × 10^8^ CFU/mL (OD_600nm_ = 0.3) with SDW. Watermelon seeds (cv. Ruixin, *n* = 24) were soaked in the adjusted bacterial cell suspensions and SDW (negative control) respectively for 4 h with continuous agitation at 28 °C and 60 rpm. Inoculated seeds were then air-dried for 24 h and planted in potting mix (PINDSTRUP sphagnum, nutritive soil, vermiculite, and perlite) in plastic pots (Guangdahengyi, Beijing, China). Pots were placed in a growth chamber for 14 days under the conditions described in 4.2. BFB symptoms were visually assessed at 14 dpi.

#### 4.4.2. Watermelon Spray Inoculation Assays

Watermelon seedling leaves that were 3-weeks-old (cv. Jingxin#3) were spray-inoculated with bacterial cell suspension (200 mL OD_600nm_ = 0.3 Aac5-pBBR, Δ*hrpE*-pBBR, and Δ*hrpE*-comp, and 200 mL SDW as the negative control), and disease index was used to quantify effects of treatment on inoculated seedlings. Inoculated seedlings were covered with transparent plastic bags and placed in the growth chamber for 30 days. Symptoms were evaluated at 10, 20, and 30 dpi, and the disease severity was assessed according to previously described methods with slight modifications [72]. In short, the severity was classified at 6 levels, 0, 1, 3, 5, 7 and 9. ‘0’ represents no symptoms, while ‘1’ = 25% necrosis of leaves, ‘3’ = 50%, ‘5’ = 75%, ‘7’ = 100% and ‘9’ = complete death of the seedlings. The disease index for each sample was calculated based on the formula DI = ∑ (disease scale × number of seedlings in each disease scale) × 100%/∑ (Total number of seedlings in each treatment × 9).

#### 4.4.3. *N. benthamiana* Inoculation Assays

The 3~4 -week-old *N. benthamiana* leaves were syringe-infiltrated with Aac5-pBBR, Δ*hrpE*-pBBR, and Δ*hrpE*-comp suspensions at OD_600nm_ = 0.3 (SDW as negative control) and incubated in a growth chamber. Each treatment group has six replicates [46].

### 4.5. HR on Non-Host Tobacco

#### 4.5.1. Qualitative Determination

Aac5-pBBR, Δ*hrpE*-pBBR, and Δ*hrpE*-comp suspensions were injected into the leaves of 3-week-old tobacco (*N. tabacum* var. *Samsun* NN) at OD_600nm_ = 0.3 for the HR assay [73]. Suspensions were injected into interspaces between leaf veins on the same leaf, with SDW as the negative control. The cell necrosis on leaf tissues was observed visually at 48 hpi.

#### 4.5.2. Quantitative Determination

HR response was quantified by measuring electrolyte leakage induced by *A. citrulli* according to a previous method with slight modifications [74]. Briefly, Aac5-pBBR, Δ*hrpE*-pBBR, and Δ*hrpE*-comp suspensions (OD_600nm_ = 0.3) were infiltrated in non-host tobacco *Samsun* leaves, with SDW as the negative control. Six leaf disks (0.7 cm in diameter) from each treatment were harvested at 2, 4, 6, 8, 10, and 12 hpi. Disks were incubated in 50 mL centrifuge tubes with 10 mL SDW respectively (one tube per disk), and continuously shaken for 30 min before measuring. Liquid conductivity was measured with a DDSJ-318 conductivity meter (REX, Shanghai INESA Scientific Instrument Co., Ltd., Shanghai, China).

### 4.6. Growth Ability In Vivo and In Vitro

#### 4.6.1. Growth Ability In Vitro

To determine the effects of *hrpE* on the growth ability of *A. citrulli* in vitro, each strain at OD_600nm_ of 0.3 was diluted 100-fold with KB/XVM2 broth in a well of a 100-well plate, with the corresponding medium as the negative control. The plate was incubated at 28 °C with continuous shaking at 220 rpm, and optical density at 600 nm was measured every 2 h for 72 h in a Bioscreen C Chamber (FP-1100-C; Oy Growth Curves Ab Ltd., Helsinki, Finland) [61].

#### 4.6.2. Growth Ability In Vivo

To determine the effects of *hrpE* on *A. citrulli* colonization, 2-week-old watermelon cotyledons (cv. Jingxin#3, *n* = 96) were syringe-infiltrated with Aac5-pBBR, Δ*hrpE*-pBBR, and Δ*hrpE*-comp suspensions (OD_600nm_ = 0.3) and incubated in a growth chamber. Population levels of *A. citrulli* in watermelon cotyledons were quantified as previously described with slight modifications [75]. Cotyledons were injected with 1 mL of inoculum, and photographed at 24, 48, 72, and 96 hpi using an EOS 70D camera (Canon, Canon (China) Co., Ltd., Beijing, China). As negative controls, plants were injected with SDW. Six leaf disks (5 mm diameter) per treatment were collected and homogenized in 600 μL SDW. Samples were mixed with lysate for 30 min, and 100 μL of supernatant was serially diluted with sterile water. 10 μL solution from each gradient concentration was plated on KB + Amp medium plates and incubated at 28 °C for 48–72 h. Colonies on plates were counted and bacterial population levels in cotyledons were calculated. The experiment was replicated three times independently.

### 4.7. Biofilm Formation Assay

The effects of *hrpE* on biofilm formation in *A. citrulli* were qualitatively and quantitatively measured according to previously described methods with slight modifications [72]. Overnight cultures of Aac5-pBBR, Δ*hrpE*-pBBR, and Δ*hrpE*-comp strains in KB and XVM2 broth were adjusted to 3 × 10^8^ CFU/mL with the corresponding medium, 1 mL of each suspension was placed in 24-well polystyrene cell culture plates (Costar 3524, Corning, NY, USA), and incubated in an artificial incubator at 28 °C for 48 h statically (with the corresponding medium as a negative control). Suspensions were fixed in an 80 °C oven for 30 min (MEMERT, Schwabach, Germany), and plates were washed with SDW. 1.5 mL of 0.1% crystal violet was added to each well and incubated at room temperature for 30 min. The liquid was discarded and washed with SDW three times. Plates were dried at 37 °C and photographed. 2 mL of 95% ethanol was added to the biofilm formed by tested strains, and the elute was measured with a spectrophotometer to analyze the strains’ biofilm-forming properties quantitatively. Each treatment has six replicates, and the experiment was conducted three times.

### 4.8. Swimming and Twitching Motility Assays

The effects of *hrpE* on swimming and twitching motility of *A. citrulli* were measured according to the methods described in Wang et al. (2016) [72] and all experiments were conducted three times.

#### 4.8.1. Swimming Motility Assay

10 µL of Aac5-pBBR, Δ*hrpE*-pBBR, and Δ*hrpE*-comp suspensions were incubated on the centers of basal medium plates (0.03% yeast extract and tryptone) with 0.3% agar at 28 °C for 72 h. The diameter of each colony was measured, and each treatment has ten replicates.

#### 4.8.2. Twitching Motility Assay

Strains Aac5-pBBR, Δ*hrpE*-pBBR, and Δ*hrpE*-comp were streaked on KB plates and incubated at 28 °C for 72 h. Corrugated tracks or halos around colonies were observed with an Olympus IX83 microscope (OLYMPUS, Tokyo, Japan). The ratio of the halo diameter and inner circle diameter was used to measure twitching motility, and each treatment was replicated three times.

### 4.9. Analysis of Aac5 Growth in Watermelon and Tobacco Leaves Pre-Treated with HrpE

To determine whether the HrpE could help hosts resist the infection of *A. citrulli*, the growth ability of Aac5 in watermelon and *N. benthamiana* leaves pre-treated with HrpE was assessed [30,31]. 4 to 5-week-old *N. benthamiana* seedlings and 2-week-old watermelon seedlings were used in these assays. All bacterial suspensions were adjusted to OD_600nm_ = 0.3, and the *A. tumefaciens* strains were exposed to 3 h dark treatment before injection. All experiments were conducted three times.

#### 4.9.1. Construction of Transient Expression Strains

The full length of *hrpE* was cloned and fused with the pYBA1132 vector [76]. The recombinant plasmid *hrpE*-pYBA1132 was transformed into DH5α and verified by sequencing. Then, the correct plasmid was extracted from DH5α and transformed into *A. tumefaciens* GV3101 [77] to obtain the *hrpE*-pYBA1132-GV3101 strain, which was PCR verified with the primer pair *hrpE*-1132-F/R.

#### 4.9.2. Assays on *N. benthamiana*

The *hrpE*-pYBA1132-GV3101 and pYBA1132-GV3101 suspensions cultured in LB broth were spun down and resuspended with buffer solution (50 mL SDW containing acetosyringone (AS) 100 μL, 10 mM MgCl_2_ 500 μL, and 2-(N-morpholino) ethanesulfonic acid (MES) 500 μL) [46]. *N. benthamiana* leaves were pre-injected with *hrpE*-pYBA1132-GV3101 suspensions, with the pYBA1132-GV3101 strain serving as the negative control and the buffer solution as the blank control. Suspensions with 3 × 10^4^ CFU/mL Aac5 (resuspended in buffer solution) were injected into HrpE-pre-injected leaves. The Aac5 inoculated plants were cultured in a growth chamber for 48 h. Six leaf disks (5 mm in diameter) were sampled for each treatment at 24 and 48 h after inoculation with Aac5. Aac5 population levels in pre-treated *N. benthamiana* leaves were assessed according to methods described in Section 4.6.2.

#### 4.9.3. Watermelon Seedling Assays

The *hrpE*-pYBA1132-GV3101 and pYBA1132-GV3101 suspensions were spun down and resuspended with 10 mM MgCl_2_ solution before injection. Full watermelon cotyledons were pre-injected with *hrpE*-pYBA1132-GV3101, using the pYBA1132-GV3101 suspensions as the negative control and the 10 mM MgCl_2_ solution as the blank control. Aac5 suspensions of 3 × 10^4^ CFU/mL density were injected into pre-injected leaves at 48 hpi. Six leaf disks (5 mm diameter) per treatment were sampled every 24 h until 96 h. Aac5 growth in pre-treated watermelon cotyledons was assessed according to methods described in Section 4.6.2.

### 4.10. Subcellular Localization of HrpE in N. benthamiana

The *hrpE*-pYBA1132-GV3101 and pYBA1132-GV3101 strains were transiently expressed in 3~4-week-old *N. benthamiana* leaves at OD_600nm_ = 0.3. At 30~36 hpi, inoculated leaves were visualized on a confocal laser microscope (Carl Zeiss LSM 980, Jena, Germany) [44]. The control for plasma membrane localization (PM) has a red fluorescent protein tag (RFP) [78]. Likewise, the control for cell nucleus localization is vector H2B-RFP which also carries an RFP tag [79]. All experiments were conducted three times.

### 4.11. ROS Production Assays

To determine the role of HrpE in inducing ROS production in hosts, qualitative assays with *N. benthamiana* and watermelon seedlings, and quantitative assays with *N. benthamiana* leaves were carried out according to Zhang et al. [45] with slight modifications. Five-week-old *N. benthamiana* seedlings and 2-week-old watermelon seedlings were used. All bacterial suspensions were adjusted to OD_600nm_ = 0.3, and the *A. tumefaciens* strains were exposed to darkness for 3 h before injection. All experiments were conducted three times.

#### 4.11.1. ROS Production in *N. benthamiana* Leaves

- Qualitative determination of ROS induced by protein

*N. benthamiana* leaves were fully injected with the *hrpE*-pYBA1132-GV3101 suspension, pYBA1132-GV3101 suspension, or buffer solution (described in Section 4.9.2) and cultivated for 48 h in a growth chamber. Leaves were collected at 48 hpi, washed by SDW, and placed in DAB staining solution (1 mg/mL DAB, pH 3.8) at room temperature for 8 h. Leaves were then removed from the DAB solution and washed with SDW three times. Leaves were boiled in 95% ethanol in a 99.99 °C water bath for 30 min until leaves were completely discolored. Discolored leaves were stored in 75% ethanol and photographed [80]. Each treatment has six replicates.

- Qualitative determination of ROS induced by strains

The Aac5-pBBR, Δ*hrpE*-pBBR, and Δ*hrpE*-comp suspensions in KB broth were spun down and then resuspended with the buffer described in Section 4.9.2. Suspensions were injected with a syringe into *N. benthamiana* leaves. The buffer solution was used as the negative control. Injected plants were cultivated for 48 h. Follow-up treatment was the same as described above.

- Quantitative determination of ROS induced by protein

*N. benthamiana* leaves were injected with the *hrpE*-pYBA1132-GV3101 and pYBA1132-GV3101 suspensions and cultivated in a growth chamber for 48 h, with the buffer solution described in Section 4.9.2 as the negative control. Thirty-six leaf disks (4 mm in diameter) were collected from each treatment and placed in a 96-well white polystyrene microplate (Costar 3922, Corning) with 100 μL SDW for 8 h at 28 °C. Then, the SDW was discarded, and a 100 μL solution (containing 100 nM flg22, 20 mg/mL horseradish peroxidase, and 100 mM luminol) was added to each well. Luminescence was immediately recorded using a chemiluminescence detector (Promega GloMAX-Multi+, Madison, WI, USA) for 60 min [80,81].

- Quantitative determination of ROS induced by strains

Aac5-pBBR, Δ*hrpE*-pBBR, and Δ*hrpE*-comp suspensions were injected into leaves, and the buffer solution served as the negative control. Plants were cultivated for 48 h, and ROS induced by strains was also measured quantitatively as described above.

#### 4.11.2. ROS Production in Watermelon Cotyledons

The amount of ROS produced in watermelon cells cannot be quantified due to the thickness of watermelon cotyledons. Therefore, only qualitative experiments were carried out, which were divided into two parts: ROS production induced by transient expression protein HrpE and induced by Aac5-pBBR, Δ*hrpE*-pBBR, and Δ*hrpE*-comp strains. The tested strains were centrifuged and resuspended with 10 mM MgCl_2_ solution before injection, using the 10 mM MgCl_2_ solution as the negative control.

- Induced by protein

The *hrpE*-pYBA1132-GV3101 and pYBA1132-GV3101 suspensions were individually injected into watermelon cotyledons, and inoculated plants were cultivated in a growth chamber. Leaves were collected at 48 hpi, washed with SDW, and placed in a DAB staining solution for 8 h. Next, they were washed with SDW three times, leaves were discolored, and photographed. Each treatment has six replicates.

- Induced by strains

The Aac5-pBBR, Δ*hrpE*-pBBR, and Δ*hrpE*-comp suspensions were each injected into watermelon cotyledons and plants were incubated for 48 h. ROS quantification was performed as described above.

### 4.12. Callose Staining

To determine the effects of HrpE in inducing callose production and deposition in hosts, the following assay was performed based on previously described methods [31,82,83] with slight modifications. Assays were carried out on 3 to 4-week-old *N. benthamiana* leaves, because watermelon cotyledons were too thick to be observed with a microscope. Strains used in these assays were adjusted to 3 × 10^8^ CFU/mL (OD_600nm_ = 0.3), centrifuged at 5000 rpm for 10 min, and resuspended with the buffer solution described in Section 4.9.2. The *A. tumefaciens* strains were treated in darkness for 3 h before injection. All experiments were replicated three times. Software Image J [31] was used to calculate the total area of callose in each digital photograph.

#### 4.12.1. Strain Induction

The Aac5-pBBR, Δ*hrpE*-pBBR, and Δ*hrpE*-comp suspensions were injected into *N. benthamiana* leaves, and the buffer solution served as control. At 20 hpi, 15 to 20 leaf disks (1 cm in diameter) were collected from each treatment, placed in 50 mL tubes with destaining solution (volume ratio of phenol: lactic acid: glycerin: SDW: ethyl alcohol was 1:1:1:1:8), and boiled in a water bath for 30 min at 65 °C. When the leaves were completely discolored, leaves were transferred to the new destaining solution, and stored at room temperature for 24 h. Leaves were then washed with 50% ethyl alcohol and SDW successively, and stained in aniline blue (0.04% aniline blue in 150 mM K_2_HPO_4_ solution) for 30 min. The stained leaves were stored in 50% glycerin and observed by UV fluorescence microscopy. A minimum of 15 photographs were taken for each treatment.

#### 4.12.2. Protein Induction

*N. benthamiana* leaves were injected with *hrpE*-pYBA1132-GV3101 and pYBA1132-GV3101 suspensions and incubated in a growth chamber for 20 h. The buffer solution served as a control. Follow-up treatment was the same as described above, and at least 30 different photographs were collected for each treatment.

### 4.13. DNA/RNA Extraction and RT-qPCR

The total DNA of bacterial strains was extracted using the Bacterial Genome DNA Extraction Kit (DP302, TIANGEN, Beijing, China). Total RNA of strains was isolated with the reagent TRIzol (Invitrogen, Waltham, MA, USA), and total RNA of collected plant leaves was extracted using the RNA Easy Fast Plant Tissue RNA Rapid Extraction Kit (DP452, TIANGEN, Beijing, China). The FastKing One Step Genome Removal and cDNA Strand Synthesis Premixed Reagent Kit (KR118, TIANGEN, Beijing, China) was used to reverse-transcribe RNA into cDNA [39].

RT-qPCR was performed using the SuperReal PreMix Plus (SYBR Green) Kit (FP205, TIANGEN, Beijing, China) on an Applied Biosystems 7500 instrument (ABI, Waltham, MA, USA). The primers used for RT-qPCR are listed in Table S1. When investigating the relative expression difference of PTI-marker genes (*NbPti5*, *NbAcre31*, *NbGras2*) [21] in *N. benthamiana* leaves injected with *hrpE*-pYBA1132-GV3101 and pYBA1132-GV3101, the elongation factor gene *NbEF1α* was used as a reference gene [84]. Each sample was tested six times and the relative expression of genes was calculated according to the 2^−ΔΔCT^ method [85].

### 4.14. Statistical Analyses

All data were analyzed with the normality test and homogeneity of variance test. Data were analyzed with one-way or two-way analysis of variance (ANOVA), Bonferroni-correction, and Tukey–Kramer’s honestly significant difference tests. The RT–qPCR data were analyzed with independent sample t-tests. Statistical analyses were conducted and graphed using GraphPad PRISM 7.0 software (GraphPad, Inc., La Jolla, CA, USA). Differences in results with *p* values less than 0.05 were considered significant.

## 5. Conclusions

In this study, we analyzed the function of HrpE from both disease-causing and disease-resistant perspectives. On the one hand, HrpE is essential for the pathogenicity of *A. citrulli*. Besides as a T3SS component, it affected other pathogen activities such as growth ability, motility, and biofilm formation, performing functions beyond a structural protein. On the other hand, HrpE could trigger host PTI responses such as ROS burst, callose deposition, and expression of PTI marker genes, and thus function in plant disease resistance. These findings revealed the importance of HrpE in the *A. citrulli*-hosts interactions, and the potential to be developed as a biopesticide. In the future, we will further analyze the interaction mechanism between HrpE and host, and provide a theoretical basis for the application of HrpE.

## Data Availability

Not applicable.

## Supplementary Materials

The following supporting information can be downloaded at: https://www.mdpi.com/article/10.3390/ijms23169144/s1.
