# Supplementary material for: Essential Acidovorax citrulli Virulence Gene hrpE Activates Host Immune Response against Pathogen
Source: Int J Mol Sci. 2022 Aug 15;23(16):9144. doi: 10.3390/ijms23169144 (PMC9409176; doi:10.3390/ijms23169144)
Supplement: Supplementary file 1 [file ijms-23-09144-s001.zip › ijms-1858320-supplementary.pdf]

Supplementary Materials

Supplementary Materials: The following supporting information can be downloaded at: https://www.mdpi.com/article/10.3390/ijms23169144/s1. Table S1: Primers used in this study; Table S2: Bacterial strains and plasmids used in this study; Figure S1: Multiple Sequence alignment of the amino acid sequences of HrpE proteins in AAC00-1 and Aac5.

Table S1. Primers used in this study

Primers	Description	Sequences (5'-3', restriction enzyme sites are underlined)	Restriction enzyme	Product length/bp	Source
WFB1	Verify whether the strain is *A. citrulli.*	GACCAGCCCACACTGGGAC	-	360	This study
WFB2		CTGCCGCACTCCAGCGA	-		
Kan^r-F	Verify whether the strain has kanamycin resistance.	CTGGACAAGGGAAAACGCA	-	700	This study
Kan^r-R		AGCCCCTGATGCTCTTCGT	-		
M13F	Sequencing primer. Verify whether the fragments have been inserted into vector pBBR1MCS-2.	TGTAAAACGACGGCCAGT	-	-	This study
M13R		CAGGAAACAGCTATGACC	-		
18F	Sequencing primer. Verify whether the fragments have been inserted into vector pK18*mobsacB.*	CAGGAAACAGCTATGAC	-	-	This study
18R		GTAAAACGACGGCCAGT	-		
hrpE-1F	Amplifying the upstream fragment of *hrpE.*	TGACATGATTACGAATTCGAACGGCTGGAGGATGGC	*Eco*R I	337	This study
hrpE-1R		GCATCGTCATCCTCCCGGTCAGCATGTGCGGCCCT	-		
hrpE-2F	Amplifying the downstream fragment of *hrpE*	GAGGGCCGCACATGCTGACCGGGAGGATGACGATGC	-	447	This study
hrpE-2R		GTCGACTCTAGAGGATCCACGGGCACCGAGAGCACT	BamH I		
hrpE-UD-F	Amplifying the upstream and downstream fragments of *hrpE,* to verify whether the gene *hrpE* has been deleted from genome.	CCGTCTCCCCCGAAGTGG	-	1 297/463	This study
hrpE-UD-R		GAGCCGAAGGGCGAGAGA	-		
hrpE-HB-F	Amplifying the fragments of *hrpE* ORF including its native promoter.	ACCGGGCCCCCCCTCGAGCCTGCCGACTCCGTGGG	Xho I	1 383	This study
hrpE-HB-R		GGCCGCTCTAGAACTAGTGGTTCCGTTCACGTCATGC	Spe I		

hrpE-JC-F	Amplifying the fragments of *hrpE*, to detecte the gene *hrpE* in strains.	GAATTCGAAACCGCCACC	-	734	This study
hrpE-JC-R		TCGTCCTGTCCATCGTCCT	-		
hrpE-1132-F	Amplifying the fragments of *hrpE* with linkers.	CGCTCTAGAACTAGTGGATCCATGCTGA TCTGGTCTTCTCCC		873	This study
hrpE-1132-R		GGGCCCCCCCTCGAGGTCGACTGCATCG TCATCCTCCCG			
NbEF1α-F	For detecting the *N. benthamiana* reference gene *NbEF1α* mRNA in qPCR assay.	AAGGTCCAGTATGCCTGGGTGCTTGAC	-	90	[84]
NbEF1α-R		AAGAATTCACAGGGACAGTTCCAATACC A	-		
NbPti5-F	For detecting the *N. benthamiana* PTI marker gene *NbPti5* mRNA in qPCR assay.	CCTCCAAGTTTGAGCTCGGATAGT	-	154	[21, 84]
NbPti5-R		CCAAGAAATTCTCCATGCACTCTGTC	-		
NbAcre31-F	For detecting the *N. benthamiana* PTI marker gene *NbAcre31* mRNA in qPCR assay.	AATTCGGCCATCGTGATCTTGGTC	-	80	[21, 84]
NbAcre31-R		GAGAAACTGGGATTGCCTGAAGGA	-		
NbGras2-F	For detecting the *N. benthamiana* PTI marker gene *NbGras2* mRNA in qPCR assay.	TACCTAGCACCAAGCAGATGCAGA	-	99	[21, 84]
NbGras2-R		TCATGAGGCGTTACTCGGAGCATT	-		

Table S2. Bacterial strains and plasmids used in this study

Strains and plasmids	Characteristics	Reference
Strains		
Acidovorax citrulli		
Aac5	Wild-type strain from group II, Amp^r	[8]
Aac5-pBBR	Aac5 containing pBBR1MCS-2; Amp^r, Kan^r	This study
Δ*hrpE*	*Aave_0464 (hrpE)* mutant strain, Amp^r	This study
Δ*hrpE*-pBBR	Δ*hrpE* containing pBBR1MCS-2; Amp^r, Kan^r	This study
Δ*hrpE*-comp	Δ*hrpE* complementary strain, Δ*hrpE* containing pBBR-*hrpE*, Amp^r, Kan^r	This study
Escherichia coli		
E. coli DH5α	*supE44 ΔlacU169* (Φ80*lacZ ΔM15*) *hsdR17 recA1 endA1 gyrA96 thi-1 relA1*	[69]
Agrobacterium tumefaciens		
GV3101	*C58 (rif^r) Ti pMP90 (pTiC58DT-DNA) (gent^r /strep^r) Nopaline*	[77]
pYBA1132-GV3101	GV3101 containing pYBA1132; Kan^r, Rif^r	This study
hrpE-pYBA1132-GV3101	GV3101 containing *hrpE*-pYBA1132; Kan^r, Rif^r	This study

PM	A plasma membrane marker, carrying red fluorescent protein (RFP) tag	[78]
H2B-RFP	A nuclear marker, carrying RFP tag	[79]
Plasmids		
pK18*mobsacB*	Cloning and suicide vector with a *sacB* gene for mutagenesis, Kan^r	[68]
pK18-*hrpE*-UD	pK18*mobsacB* carrying 447 bp and 337 bp upstream and downstream sequences of the *Aave_0464* gene, Kan^r	This study
pBBR1MCS-2	Broad-host range expression vector, Kan^r	[71]
pBBR-*hrpE*	pBBR1MCS-2 carrying a 1,383 bp fragment containing *hrpE* and its native promoter, Kan^r	This study
pRK600	Helper strain in tri-parental mating, Cm^r	[70]
pYBA1132	A plant expression vector carrying a fluorescent gene *eGFP* (Enhanced green fluorescent protein, eGFP) and containing a 35S promoter; Kan^r	[76]
hrpE-pYBA1132	pYBA1132 carrying an 873 bp fragment containing *Aave_0464* ORF with no termination codon, Kan^r	This study

Amp^r, Kan^r, Cm^r and Rif^r indicate resistance to ampicillin, kanamycin, chloramphenicol, and rifampicin respectively.

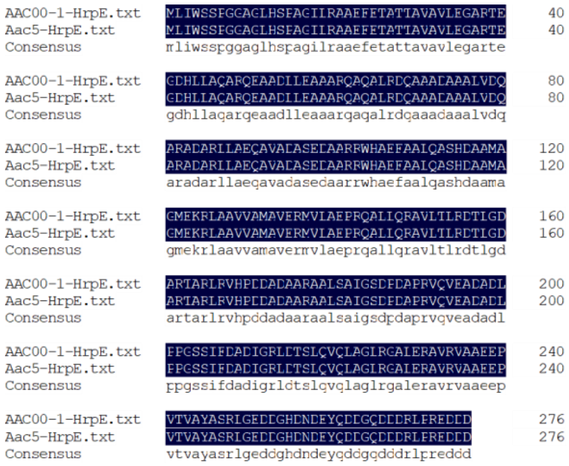

Figure S1. Multiple Sequence alignment of the amino acid sequences of HrpE proteins in *Acidovorax citrulli* strains AAC00-1 and Aac5. HrpE in strain Aac5 was 100% identical to the sequences of *Aave_0464* (*hrpE*) from AAC00-1 (GenBank accession number CP000512.1).
